# Social diversity, parenting, and child development promotion: reflections on nursing care

**DOI:** 10.1590/1980-220X-REEUSP-2025-0208en

**Published:** 2025-12-01

**Authors:** Carolina Beltreschi Bardivia, Maria de La Ó Ramallo Veríssimo, Elsa Maria de Oliveira Pinheiro de Melo, Débora Falleiros de Mello

**Affiliations:** 1 Universidade de São Paulo, Escola de Enfermagem de Ribeirão Preto, Programa de Pós-Graduação Enfermagem em Saúde Pública, Ribeirão Preto, SP, Brazil.; 2 Universidade de São Paulo, Escola de Enfermagem, São Paulo, SP, Brazil.; 3 Universidade de Aveiro, Escola Superior Saúde de Aveiro, Portugal.; 4 Universidade de São Paulo, Escola de Enfermagem de Ribeirão Preto, Ribeirão Preto, SP, Brazil.

**Keywords:** Child Care, Child Development, Caregivers, Parenting

## Abstract

**Objective:**

To list reflective elements on child care in the face of contemporary dilemmas involving parenting and social diversity to promote child development and its implications for nursing.

**Method:**

Reflective study based on the theoretical perspective of practical wisdom, supported by the understanding of practical knowledge involving choices in the face of everyday contingencies.

**Results:**

Reflective elements were listed on parental choices and possible contemporary dilemmas regarding social diversity related to care provided by someone of another skin color, another sexual orientation, another religion, and male figures. Practical knowledge related to parental choices and supposed resistance to other people's abilities to care for children suggests that reflections on the implications for parenting and the promotion of child development should be expanded.

**Conclusion:**

Practical wisdom contributes to analyzing and considering aspects of care, bringing important elements for nursing to assist in parental choices that encompass social diversity in childhood, considering the avoidance of discrimination, prejudice, and hostility, to value opportunities to promote full child development.

## INTRODUCTION

The care provided to children by their primary caregivers is crucial to promoting child development, with an emphasis on responsive interaction that provides affection and learning, especially during the first years of life^(1)^. In our reality, primary care is provided mainly within the family, by mothers, fathers, or people who exercise parental roles. The evaluation of the effectiveness of parental interventions in child development indicates that promotional actions are particularly important in the first three years of life, when the developing brain is most sensitive to experiences and the environment^(1)^.

Parenting is understood as the set of strategies carried out by caregivers with a parental role, with the purpose of promoting the child’s development, ensuring their safety and providing adequate stimuli^(2)^.

Parental caregivers have many tasks to perform in providing care and understanding information that may be complex, new, specific, or uncertain. It is essential to have reference people who can support parental knowledge and practices regarding child development and its benefits for comprehensive child care^(3)^.

In childcare, non-parental caregivers are also extremely important, including family members, nannies and daycare providers, who may be primary or secondary caregivers^(4)^. Promoting the integral development of children includes aspects of socialization, involving learning to live among peers and in the community, forms of interaction, empathy, respect and otherness, optimizing children’s development trajectories^(5)^.

In this process, it is necessary to pay attention to group belonging, exposure to diversity, and plural social interactions. Parental and non-parental care may contain implications of social diversity issues, including discrimination related to racism^(6)^, hostility related to diversity by sexual orientation^(7)^, obstacles to the acceptance of different religions^(8)^ and difficulties in greater involvement of the male figure in childcare^(9)^, which have been highlighted in different social contexts for their repercussions on children’s development. In particular, pre-school education institutions must ensure social and cognitive stimulation in a safe environment and with quality in the activities provided^(10)^.

In view of scientific evidence that highlights the importance of care provided to children, with an emphasis on responsive interaction and the role of positive parenting, and also in the face of contemporary dilemmas in dealing with differences, discrimination and intolerance, as well as the need to increase inclusive actions, this study seeks to discuss aspects related to parenting and social diversity and their possible consequences for the promotion of child development. Thus, the objective is to list reflective elements on child care in the face of contemporary dilemmas involving parenting and social diversity to promote child development and its implications for nursing.

## THEORETICAL-METHODOLOGICAL FRAMEWORK

Reflective study based on the perspective of practical wisdom^(10)^, seeking to understand practical knowledge involving parenting and social diversity in favor of child development promotion. In this regard, care is brought to reflection with a focus on social diversity in relation to the care of children carried out by someone of another skin color, another sexual orientation, another religion and/or by male figures.

In this study, the perspective of practical wisdom is addressed for nursing care in child health, highlighting the importance of being in line with contextualized and shared realities between parental caregivers and professionals, and seeking to value experiences, choices and decision-making for understanding and expanding health care^(11)^.

Practical wisdom is a concept originating from practical philosophy, within the scope of Aristotelian knowledge about the rationalities of knowledge^(10)^. The plan of practical wisdom involves a contingent specificity, that is, it deals with eventuality, uncertainties, events and human experiences, recognizing that the interconnected aspects are not perennial, causal and universal^(10)^. In this respect, it constitutes a space of knowledge with an outlet for the emergence of common or divergent interests, values and possibilities for interaction and dialogue.

The theoretical perspective of practical wisdom involves practical knowledge linked to the processes of choice (being able to choose, needing to choose, needing to know and finding the good in each situation experienced), and the concept of contingent as its essence^(10)^. Thus, it is also a set of knowledge that deals with less certainty and determination, and seeks to build understanding of aspects of life, experiences and choices in the face of the various contingencies of everyday life.

Practical knowledge and its interfaces with technical-scientific knowledge refer to a movement of reconstruction and articulation of different aspects that make up a theme or a situation, and that can assist the process of human choices^(10)^. In the case of child health care and development and its relationship to parenting, parental choices are very important. To this end, communication, language, listening, and dialogue between individuals are vital to fostering good care choices and critically reflecting on concerns and dilemmas of perceptions and perspectives, contributing to greater clarity toward good practices.

In the field of promoting child development, professionals can base themselves on practical wisdom, that is, paying attention to the experiences of parental caregivers and their choices regarding their children’s care needs, implying responsibility for pertinent caregiving attitudes.

## PARENTING AND CHILD DEVELOPMENT PROMOTION: A GLANCE AT SOCIAL DIVERSITY

The set of experiences that a child goes through in the first years of life makes up a repertoire of great relevance in brain formation and in the construction of learning and motor, cognitive, emotional, and social skills. Therefore, all interactions and environments must act synergistically with adequate care and stimuli to provide good development^(1)^.

The horizons of parental and non-parental care play a significant role in the lives of children and, particularly, the situations of those who care for the child and the environment in which the child is inserted in the first years of life need to compose the dialogue with their families and encourage good parental choices. When child care is viewed from the perspective of ensuring their full development, non-parental care provided by other people also gains relevance. Along this path, opportunities to interact with someone of a different skin color, sexual orientation, religion or male figure are important.

Regarding the aspects of skin color and child development, a study discusses the parental role in addressing prejudices^(6)^. It points out that children may begin to exhibit racial prejudices in the early stages of childhood development, making it necessary to prevent and reduce aspects that may involve prejudice. Discrimination issues related to racism can be a barrier to health care. A study found that racially biased parental attitudes denying white privilege were negatively related to children’s sympathy for Black victims, addressing potential ways that race-based children’s sympathy and compassion might be shaped^(12)^. Babies who grew up in more diverse communities showed a greater ability to recognize faces of other ethnicities, compared to those who lived in less diverse communities^(13)^. Thus, exposure to a variety of faces from early childhood is suggested to play a critical role in lifelong acceptance of racial diversity.

Regarding aspects of sexual orientation, issues involving sexual diversity can trigger a set of attitudes of hostility, marginalization, and exclusion. In childcare, it is important to discuss sexual orientation, parenting, and child well-being^(7)^. A study emphasizes that assumptions should not be carried out based on people’s sexual orientation, whether they are better suited than others to care for children^(7)^.

Another aspect worth highlighting concerns the obstacles to accepting different religions, with religious intolerance being able to negatively affect human development and self-esteem^(8)^. Paying attention to the connections between religion and specific cultural attitudes, for example towards immigrants, atheists and minority religious groups, becomes important for the inclusion of interdisciplinary and person-centered approaches^(8)^. The way families and schools approach coexistence with different religions can influence the acceptance or rejection of different beliefs, and the way educators deal with religious diversity contributes to the promotion of respectful coexistence^(14,15)^.

Regarding the active presence of the male figure in childcare, the implications regarding helping boys understand that care is not the exclusive responsibility of women and girls is very important, allowing them to understand that men can be caregivers, which contributes to the construction of more balanced emotional relationships^(16)^. This way, the deconstruction of stereotypes promotes a more egalitarian and collaborative view of men and women^(16)^.

Fathers who want to be more involved in childcare may face cultural barriers, a lack of resources, and inadequate support, which highlights the need to demystify the image of traditional fatherhood. Consequently, many men continue to be socialized with the expectation of becoming the financial provider, and the mother is seen as the primary caregiver for the child^(17)^. Childcare seen as a secondary function can perpetuate traditional roles of strength and authority, and this stigma can create insecurity and hinder fathers’ full parental involvement in childcare^(18)^.

In early childhood education, the male figure is seen as beneficial, as it promotes a plural formation, in addition to playing a crucial role in the integral development of the child, especially related to the construction of identity and the development of cognitive and socio-emotional skills^(19)^. In this regard, expanding the potential for male involvement in the daily care of children contributes to encouraging a gender-transformative approach^(9)^.

The opportunities afforded by children’s intergroup gender attitudes have implications for peer behavior, as exemplified in a study of 4- and 5-year-olds from diverse backgrounds (Mexican, Chinese, Dominican, and African-American), in which positive same-sex attitudes were associated with knowledge of gender stereotypes, and in contrast, other-gender attitudes were associated with cognitive versatility (stereotype flexibility, gender consistency), and other-gender attitudes predicted prejudiced behavior^(20)^. Early learning about gender categories is suggested to shape young children’s attitudes, with consequences for intergroup behavior in childhood^(20)^.

In situations of non-parental care in childhood, when the care is provided by someone of a different skin color, sexual orientation, religion or male figure, attention must be paid to the relationship between parental and non-parental caregivers. In this sense, it is essential to prioritize interactions and the care environment, so that they contribute to child development, independent of personal and social characteristics. Therefore, there is a need to focus on the involvement with the child, the potential to contribute to their development, and the perceptions and facts about future impacts. It is also important to pay attention to global cycles of poverty, inequality and social exclusion, which create difficulties in achieving human development potential^(21)^.

Such aspects are important in the understanding of practical wisdom, involving parental perceptions and choices and children full care, with the strengthening of initiatives of positive child-environment interactions and promotion of early childhood development.

Promoting equitable and inclusive environments for children, as well as approaches that encourage positive attitudes towards dealing with diversity, are essential because they provide opportunities to facilitate socialization, the development of connections, and a sense of belonging^(22)^. It is important to emphasize the need for a good composition of children’s early care environments, with timely social relationships, relevant tools to analyze the depth and breadth of relationships and social networks throughout development^(23)^. Accordingly, social categorization is constructed and has its roots in perceptive, conceptual, and social systems that emerge from an early age and undergo important developmental changes in childhood with possible repercussions throughout life^(24)^.

The basis of the human capacity to form useful social categories occurs in childhood, making it possible to guide inferences that children make from shared social characteristics and relationships^(21)^. For children, social categorization can help simplify and understand their social environment, but it has harmful consequences in the form of stereotypes, prejudices, and discrimination^(24)^. This way, the early childhood education is considered one of the ways to combat prejudices related to diversity and promote better inclusion, as long as the environments provide qualified social and cognitive stimulation^(10)^. Globally, qualitative and inclusive strategies in early childhood education have been recommended to achieve the goals of Sustainable Development Goals, including those targeted to promote child development^(22)^.

In this study, the highlighted reflective elements involve the perspectives that parental care can have in promoting child development, in which a range of parental and non-parental experiences is very relevant, with aspects that permeate choices, considerations, arguments, and reflective knowledge being vital. The dimensions of choices related to social diversity may contain obstacles and challenges or be open to breaking with intolerance, discrimination, and prejudice. In this regard, it is important to prioritize dialogue between the subjects involved to broaden understanding and redefine child care and development, which are relevant to health and nursing care in this field.

Therefore, this reflection does not intend to address or debate specific definitions for the ethnic-racial-cultural, religious, gender and sexual orientation diversity. The perspective is to reflect on nursing care in the face of challenges involving such aspects for the promotion of human development in childhood.

### Contributions to the Nursing Field

The notes regarding child care provided by other people bring important contemporary aspects to rethink nursing care involving health care in early childhood. The process of caring for children, permeated by parental choices, encompasses the theme of social diversity, and it is important to give space to reflections on the interfaces with the opportunities for the integral development of the child and the expansion of practical wisdom.

Caring for children’s health alongside parental caregivers requires skills to expand connections with practical knowledge^(11)^. The theme of social diversity in child care involves encompassing and going beyond technical success, achieving comprehensive attitudes in the care process and reconstructing understandings about the situations that families experience and how they deal with the resources available in the community.

Parental choice about who will care for the child is somewhat voluntary, but if there is more information, joint dialogue, and openness to expand thoughts, there will likely be more possibilities to support choices. In this way, it involves being more open to dealing with differences and rethinking prejudiced and discriminatory aspects. Therefore, it is necessary to create spheres of comprehensive dialogue for the diverse demands and build a step-by-step process of intercultural and plural experience.

Child health nursing care in relation to this topic requires actions that channel practical wisdom, that is, that are attentive to understanding attitudes and experiences, giving visibility to opinions and hidden dimensions, detailing desires, interests, needs, hopes and choices, as well as examining the possibilities of breaking down barriers. Thus, it turns to the ethical challenge of nurses regarding the value that health action has for parental caregivers and their children, building caring attitudes that include the subject as a participant^(11)^.

The focus on diversity, equity, and inclusion requires seeking social and cultural transformations for care, to recognize identity markers (gender, sexual orientation, race/skin color, ethnicity, religion, social class, territoriality, generation, disabilities, neurodiversity, among others), respect differences, and work toward health needs, equity, and human rights. In the early years of life, children’s interactions with the physical and social environment, including family, school, and community, play a determining role in their developmental trajectories, with long-term implications for their health, well-being, and future potential^(5)^.

There are implications for nursing teaching, research and practice with the construction of interpersonal and intercultural skills, based on scientific knowledge, active strategies and advances permeated by new situations and the encounter with differences, fundamental in the training and continuing education of nurses, who need a solid framework to deal with human beings, their health needs and unique characteristics, and for group work and interprofessional teams. The study highlights the need to incorporate the principles of diversity, equity and inclusion into nursing curricula, as well as a broader understanding of how prejudice interferes with care and health promotion, seeking to deconstruct prejudices through reflective education^(25)^.

Nursing care, with emphasis on prenatal and childcare follow-up, in consultations, home visits and educational groups, includes many elements for comprehensive care of the child’s health together with parental caregivers for positive parenting. It is also important to emphasize intersectoral actions between health, education, and social protection, which are sectors with important interfaces to enable good care management in contributing to human development.

The professional care provided in these meetings for child health care enables a series of interactions with families. For effective interactions that are responsive to families’ experiences, it is proposed that good practices should include the understanding that parental beliefs and choices are indispensable to child health nursing care, as part of a repertoire that seeks to obtain the different and the diverse and give flow for the knowledge of experiences, common or divergent interests, tensions, possibilities, and expectations to emerge.

Thus, within the scope of promoting child development, clarifying and strengthening parental choices involving the themes of diversity, equity and inclusion are part of care, so that they are more linked to the importance of the full development of children with diverse and multicultural learning and experiences from the first years of life, and that parental perceptions migrate from a list of invisibility knowledge to expand their practical knowledge.

## CONCLUSION

The study highlighted exploratory and reflective elements on parental choices related to child care, pointing out contemporary dilemmas regarding social diversity related to care provided by someone of another skin color, another sexual orientation, another religion, and male figures. Reflections on practical knowledge were emphasized for the promotion of child development, highlighting the importance of nursing in dealing with parental experiences, their choices and supposed resistance to other people’s abilities to care for the child.

The theme of social diversity involving people who care for children encompasses challenges and opportunities in the pursuit of children’s full development, that promote socialization, the development of connections, and a sense of belonging. Therefore, it is necessary to place more value on socio-emotional, cognitive, attention, memory, problem-solving and critical judgment skills, which are necessary throughout life.

Child health nursing care in the face of aspects related to social diversity needs to expand and seek to understand values and paths for satisfactory human coexistence, avoiding discrimination, prejudice, hostility and conflicts, rethinking and rebuilding relationships, actions and commitments. Thus, in comprehensive care for children’s health, understanding beliefs and choices and increasing communication are imperative in contemporary times. Research is required to understand parental caregivers’ perceptions and identify the impact of social characteristics of child caregivers on the promotion of child development.

## DATA AVAILABILITY

The entire dataset supporting the results of this study was published in the article itself.
