# Supplementary material for: Social diversity, parenting, and child development promotion: reflections on nursing care
Source: Rev Esc Enferm USP. 2025 Dec 1;59:e20250208. doi: 10.1590/1980-220X-REEUSP-2025-0208en (PMC12674172; doi:10.1590/1980-220X-REEUSP-2025-0208en)
Supplement: Translation

# Translation: Diversidade social, parentalidade e promoção do desenvolvimento infantil: reflexões ao cuidado de enfermagem

**DOI:** 10.1590/1980-220X-REEUSP-2025-0208pt

**Authors:** Carolina Beltreschi Bardivia, Maria de La Ó Ramallo Veríssimo, Elsa Maria de Oliveira Pinheiro de Melo, Débora Falleiros de Mello

**Affiliations:** 1 Universidade de São Paulo, Escola de Enfermagem de Ribeirão Preto, Programa de Pós-Graduação Enfermagem em Saúde Pública, Ribeirão Preto, SP, Brasil.; 2 Universidade de São Paulo, Escola de Enfermagem, São Paulo, SP, Brasil.; 3 Universidade de Aveiro, Escola Superior Saúde de Aveiro, Portugal.; 4 Universidade de São Paulo, Escola de Enfermagem de Ribeirão Preto, Ribeirão Preto, SP, Brasil.

**Keywords:** Cuidado da criança, Desenvolvimento infantil, Cuidadores, Poder Familiar

## Abstract

**Objetivo:**

Elencar elementos reflexivos sobre o cuidado da criança frente aos dilemas contemporâneos envolvendo a parentalidade e a diversidade social para promoção do desenvolvimento infantil e suas implicações para a enfermagem.

**Método:**

Estudo reflexivo sob a perspectiva teórica da sabedoria prática, amparada na compreensão de saberes práticos que envolvem escolhas diante de contingências do cotidiano.

**Resultados:**

Foram elencados elementos reflexivos sobre escolhas parentais e possíveis dilemas contemporâneos sobre diversidade social relativas ao cuidado exercido por alguém de outra cor de pele, outra orientação sexual, outra religião e figuras masculinas. Saberes práticos relacionados às escolhas e supostas resistências parentais às aptidões de outras pessoas para o cuidado da criança sugerem ampliar as reflexões sobre as implicações na parentalidade e na promoção do desenvolvimento infantil.

**Conclusão:**

A sabedoria prática contribui para analisar e ponderar aspectos do cuidado, trazendo elementos importantes para a enfermagem auxiliar as escolhas parentais que abrangem a diversidade social na infância, considerando evitar discriminação, preconceitos e hostilidades, para valorizar as oportunidades de promoção do desenvolvimento infantil pleno.

## INTRODUÇÃO

Os cuidados ofertados às crianças por seus cuidadores primários são cruciais para promover o desenvolvimento infantil, com ênfase na interação responsiva que proporciona afeto e aprendizado, principalmente durante os primeiros anos de vida^(1)^. Em nossa realidade, os cuidados primários são ofertados principalmente no âmbito familiar, pelas mães, pais ou pessoas que exercem a função parental. A avaliação da eficácia das intervenções parentais no desenvolvimento infantil aponta que ações de promoção são particularmente importantes nos primeiros três anos de vida, quando o cérebro em desenvolvimento é mais sensível às experiências e ao ambiente^(1)^.

A parentalidade é entendida como o conjunto de estratégias realizadas pelos cuidadores com função parental, com a finalidade de promover o desenvolvimento da criança, garantir sua segurança e proporcionar os estímulos adequados^(2)^.

Os cuidadores parentais têm muitas tarefas para exercer o cuidado e compreender informações que podem ser complexas, novas, específicas ou incertas, sendo essencial contar com pessoas de referência que possam apoiar os conhecimentos e práticas parentais sobre o desenvolvimento infantil e seus benefícios para o cuidado integral da criança^(3)^.

No cuidado das crianças, também são de extrema importância os cuidadores não parentais, incluindo familiares, babás e educadores de creches, que podem ser cuidadores primários ou secundários^(4)^. A promoção do desenvolvimento integral da criança inclui aspectos de socialização, envolvendo a aprendizagem da convivência entre pares e na comunidade, formas de interação, empatia, respeito e alteridade, otimizando as trajetórias de desenvolvimento das crianças^(5)^.

Nesse processo, é necessário atentar para o pertencimento ao grupo, a exposição à diversidade e às interações sociais plurais. Os cuidados parentais e não parentais podem conter implicações das questões de diversidade social, abrangendo a discriminação referente ao racismo^(6)^, hostilidade relacionada à diversidade por orientação sexual^(7)^, obstáculos para aceitação de diferentes religiões^(8)^ e dificuldades para maior envolvimento da figura masculina nos cuidados infantis^(9)^, as quais têm sido apontadas em diferentes contextos sociais pelas suas repercussões no desenvolvimento das crianças. Particularmente, as instituições de educação infantil devem garantir estimulação social e cognitiva em um ambiente seguro e com qualidade nas ações ofertadas^(10)^.

Diante de evidências científicas que destacam a importância dos cuidados ofertados às crianças, com ênfase na interação responsiva e no papel da parentalidade positiva, e também perante dilemas contemporâneos em lidar com o diferente, a discriminação e a intolerância, bem como a necessidade de incrementar ações inclusivas, este estudo busca discutir aspectos relativos à parentalidade e diversidade social e seus possíveis desdobramentos à promoção do desenvolvimento infantil. Assim, o objetivo é elencar elementos reflexivos sobre o cuidado da criança frente aos dilemas contemporâneos envolvendo a parentalidade e a diversidade social para promoção do desenvolvimento infantil e suas implicações para a enfermagem.

## REFERENCIAL TEÓRICO-METODOLÓGICO

Estudo reflexivo sob a perspectiva da sabedoria prática^(10)^, em busca de compreender saberes práticos envolvendo a parentalidade e a diversidade social em prol da promoção do desenvolvimento infantil. Nesse sentido, traz-se para reflexão o cuidado com foco na diversidade social em relação ao cuidado da criança exercido por alguém de outra cor de pele, outra orientação sexual, outra religião e ou por figuras masculinas.

No presente estudo, a perspectiva da sabedoria prática é abordada para o cuidado de enfermagem em saúde da criança, ressaltando a importância de estar em consonância com realidades contextualizadas e compartilhadas entre cuidadores parentais e profissionais, e em busca de valorizar experiências, escolhas e tomadas de decisão para compreensão e ampliação do cuidado em saúde^(11)^.

A sabedoria prática é um conceito originário da filosofia prática, no âmbito dos conhecimentos aristotélicos sobre as racionalidades dos saberes^(10)^. O plano da sabedoria prática envolve uma especificidade contingente, ou seja, lida com a eventualidade, as incertezas, os acontecimentos e as experiências humanas, reconhecendo que os aspectos interligados não são perenes, causais e universais^(10)^. Nessa vertente, constitui um espaço de saber com vazão para emergir os interesses comuns ou divergentes, os valores e as possibilidades de interação e diálogo.

A perspectiva teórica da sabedoria prática envolve os saberes práticos ligados aos processos de escolha (conseguir escolher, precisar escolher, necessitar saber e encontrar o bem em cada situação vivenciada) e ao conceito de contingente como sua essência^(10)^. Assim, trata-se também de um conjunto de saberes que lidam com menos certeza e determinação, e buscam construir a compreensão de aspectos da vida, das experiências e das escolhas diante das diversas contingências do cotidiano.

Os saberes práticos e suas interfaces com os conhecimentos técnico-científicos referem-se a um movimento de reconstrução e articulação de diferentes aspectos que compõem um tema ou uma situação, e que podem auxiliar o processo das escolhas humanas^(10)^. No caso dos cuidados à saúde e desenvolvimento infantil e sua relação com a parentalidade, as escolhas parentais são muito importantes. Para tanto, são vitais a comunicação, a linguagem, a escuta e o diálogo entre os sujeitos, para fomentar boas escolhas de cuidado e refletir criticamente sobre as preocupações e dilemas de percepções e perspectivas, e que contribuam para ter mais clareza rumo às boas práticas.

No campo da promoção do desenvolvimento infantil, os profissionais podem fundamentar-se na sabedoria prática, ou seja, atentar para as experiências dos cuidadores parentais e suas escolhas frente às necessidades do cuidado de suas crianças, implicando em responsabilidade por atitudes cuidadoras pertinentes.

## PARENTALIDADE E PROMOÇÃO DO DESENVOLVIMENTO INFANTIL: UM OLHAR PARA A DIVERSIDADE SOCIAL

O conjunto de experiências que a criança vivencia nos primeiros anos de vida compõe um repertório de grande relevância na formação do cérebro e na construção de aprendizagens e habilidades motoras, cognitivas, emocionais e sociais. Assim, todas as interações e ambientes devem atuar sinergicamente com cuidados e estímulos adequados para proporcionar um bom desenvolvimento^(1)^.

Os horizontes do cuidado parental e não parental exercem papel significativo para a vida das crianças e, particularmente, as situações de quem cuida da criança e do ambiente em que ela se insere nos primeiros anos de vida necessitam compor o diálogo com as famílias e favorecer boas escolhas parentais. Quando o cuidado da criança é visto na perspectiva de proporcionar o seu desenvolvimento pleno, também ganham relevância os cuidados não parentais desempenhados por outras pessoas. Nesse caminho, são importantes as oportunidades de convivência com alguém de outra cor de pele, outra orientação sexual, outra religião ou figuras masculinas.

No tocante aos aspectos sobre a cor da pele e desenvolvimento infantil, estudo discute o papel parental na abordagem dos preconceitos^(6)^. Aponta que as crianças podem começar a exibir preconceitos raciais em fases iniciais do desenvolvimento infantil, sendo necessário prevenir e diminuir os aspectos que possam envolver prejulgamentos. Questões de discriminação relacionadas ao racismo podem ser uma barreira aos cuidados de saúde. Estudo encontrou que atitudes parentais raciais com negação do privilégio dos brancos foram negativamente relacionadas com a simpatia das crianças pelas vítimas negras, abordando as formas potenciais de moldar a simpatia e compaixão das crianças com base na raça^(12)^. Bebês que cresceram em comunidades mais diversas mostraram uma capacidade mais aprimorada de reconhecer rostos de outras etnias, em comparação com aqueles que viveram em comunidades menos diversas^(13)^. Assim, a exposição a uma variedade de rostos desde tenra infância sugere desempenhar um papel crucial na aceitação da diversidade racial ao longo da vida.

Em relação aos aspectos sobre orientação sexual, as questões envolvendo a diversidade sexual podem desencadear um conjunto de atitudes de hostilidade, marginalização e exclusão. No cuidado das crianças, é importante o debate sobre orientação sexual, parentalidade e bem-estar das crianças^(7)^. Estudo enfatiza que não devem ser realizadas suposições com base na orientação sexual das pessoas, se são mais adequadas do que outras para cuidar das crianças^(7)^.

Outro aspecto a destacar refere-se aos obstáculos para aceitação de diferentes religiões, com a intolerância religiosa podendo afetar negativamente o desenvolvimento e a autoestima humana^(8)^. Atentar para as conexões entre religião e atitudes culturais específicas, por exemplo em relação aos imigrantes, ateus e grupos religiosos minoritários, ganha importância para a inclusão de abordagens interdisciplinares e centradas na pessoa^(8)^. A forma como as famílias e as escolas abordam a convivência com diferentes religiões pode influenciar na aceitação ou rejeição de crenças distintas, e a maneira como os educadores lidam com a diversidade religiosa contribui para a promoção de uma convivência respeitosa^(14,15)^.

Quanto à presença ativa da figura masculina no cuidado infantil, implicações no que se refere a ajudar meninos a compreender que o cuidado não é uma responsabilidade exclusiva das mulheres e meninas é muito importante, além de possibilitar apreender que os homens podem ser cuidadores, o que contribui para a construção de relações afetivas mais equilibradas^(16)^. Desse modo, a desconstrução de estereótipos promove uma visão mais igualitária e colaborativa entre homens e mulheres^(16)^.

A figura paterna que deseja ser mais envolvida no cuidado das crianças pode enfrentar barreiras culturais, falta de recursos e um apoio inadequado, destacando a necessidade de desmistificar a imagem da paternidade tradicional. Assim, muitos homens continuam a ser socializados com a expectativa de se tornar o provedor financeiro, e a mãe é vista como a principal responsável pela criança^(17)^. O cuidado das crianças visto como uma função secundária pode perpetuar os papéis tradicionais de força e autoridade, e esse estigma pode gerar insegurança e dificultar o envolvimento parental paterno no cuidado pleno das crianças^(18)^.

Na educação infantil, a figura masculina é vista como benéfica, pois promove uma formação plural, além de desempenhar um papel crucial no desenvolvimento integral da criança, especialmente relacionado à construção de identidade e desenvolvimento de habilidades cognitivas e socioemocionais^(19)^. Nesse sentido, ampliar o potencial de envolvimento de figuras masculinas no cuidado cotidiano da criança contribui para estimular uma abordagem transformadora de gênero^(9)^.

As oportunidades que proporcionam atitudes intergrupais de gênero das crianças têm implicações para o comportamento entre pares, a exemplo do estudo com crianças entre 4 e 5 anos de idade de diversas origens (mexicanas, chinesas, dominicanas e afro-americanas), em que as atitudes positivas em relação ao mesmo sexo foram associadas ao conhecimento dos estereótipos de gênero e, em contraste, atitudes em relação a outros gêneros foram associadas à versatilidade cognitiva (flexibilidade de estereótipos, consistência de gênero), e atitudes de outro gênero previram comportamento preconceituoso^(20)^. A aprendizagem precoce sobre as categorias de gênero sugere moldar as atitudes das crianças pequenas, com consequências para o comportamento intergrupal na infância^(20)^.

Nas situações de cuidados não-parentais na infância, quando o exercício do cuidado é realizado por alguém de outra cor de pele, outra orientação sexual, outra religião e ou figura masculina, há que ter atenção para a relação entre cuidadores parentais e não parentais. Nesse sentido, é fundamental haver prioridade para interações e ambiente de cuidado, para que contribuam para o desenvolvimento infantil, descolados das características pessoais e sociais. Portanto, há necessidade de um olhar para o envolvimento com a criança, os potenciais de contribuir para o seu desenvolvimento, e as percepções e fatos sobre impactos futuros. Também é importante atentar para ciclos globais de pobreza, desigualdades e exclusão social, que geram dificuldades para atingir os potenciais de desenvolvimento humano^(21)^.

Tais aspectos ganham importância na compreensão da sabedoria prática, envolvendo as percepções e escolhas parentais e o cuidado pleno das crianças, com o fortalecimento das iniciativas de interações positivas criança-ambiente e de promoção do desenvolvimento na primeira infância.

Promover ambientes equitativos e inclusivos para as crianças, bem como as abordagens que incentivam atitudes positivas para lidar com a diversidade são essenciais, porque configuram oportunidades de viabilizar a socialização, o desenvolvimento de conexões e um sentimento de pertencimento^(22)^. Cabe enfatizar a necessidade de uma boa composição dos primeiros ambientes de cuidado das crianças, com relações sociais oportunas, ferramentas pertinentes para analisar a profundidade e a amplitude das relações, e redes sociais ao longo do desenvolvimento^(23)^. Assim, a categorização social vai sendo construída, e tem suas raízes em sistemas perceptivos, conceituais e sociais que emergem desde tenra idade e passam por importantes mudanças de desenvolvimento na infância com possíveis repercussões ao longo da vida^(24)^.

A base da capacidade humana de formar categorias sociais úteis ocorre na infância, possibilitando orientar inferências que as crianças fazem a partir das características e relações sociais partilhadas^(21)^. Para as crianças, a categorização social pode ajudar a simplificar e compreender o seu ambiente social, mas tem consequências prejudiciais sob a forma de estereótipos, preconceitos e discriminação^(24)^. Nesse caminho, a educação infantil é considerada uma das formas de combater preconceitos relacionados à diversidade e de promover uma melhor inclusão, desde que os ambientes proporcionem estimulação social e cognitiva qualificadas^(10)^. Globalmente, estratégias qualitativas e inclusivas na educação infantil têm sido recomendadas para atingir as metas dos Objetivos de Desenvolvimento Sustentável, incluindo os direcionados à promoção do desenvolvimento da criança^(22)^.

No presente estudo, os elementos reflexivos de destaque envolvem as perspectivas que o cuidado parental pode ter na promoção do desenvolvimento infantil, em que uma gama de experiências parentais e não parentais é muito relevante, sendo vitais os aspectos que perpassam por escolhas, considerações, argumentações e saberes reflexivos. As dimensões das escolhas referentes à diversidade social podem conter obstáculos e desafios ou terem abertura para romper com a intolerância, a discriminação e os preconceitos. Nesse caminho, cabe privilegiar o diálogo entre os sujeitos envolvidos para ampliar os entendimentos e ressignificar o cuidado da criança e o seu desenvolvimento, que são relevantes para o cuidado em saúde e de enfermagem nesse campo.

Assim, esta reflexão não pretende abordar ou debater definições específicas para a diversidade étnica-racial-cultural, religiosa, de gênero e orientação sexual. A perspectiva é refletir sobre o cuidado de enfermagem em face aos desafios envolvendo tais aspectos para a promoção do desenvolvimento humano na infância.

### Contribuições para a Área da Enfermagem

Os apontamentos em relação ao cuidado da criança exercido por outras pessoas trazem aspectos contemporâneos importantes para repensar o cuidado de enfermagem envolvendo a atenção à saúde na primeira infância. O processo de cuidar das crianças, permeado por escolhas parentais, engloba a temática da diversidade social, sendo importante dar espaço às reflexões sobre as interfaces com as oportunidades do desenvolvimento integral da criança e a expansão da sabedoria prática.

Cuidar da saúde das crianças junto aos cuidadores parentais requer competências para expandir as articulações com os saberes práticos^(11)^. A temática da diversidade social no cuidado da criança implica abranger e ultrapassar o êxito técnico, alcançando atitudes compreensivas no processo de cuidar e reconstruindo os entendimentos sobre as situações que as famílias vivenciam e como lidam com os recursos disponíveis na comunidade.

A escolha parental sobre quem vai cuidar da criança é de certo modo voluntária, mas se houver mais informações, diálogo conjunto e abertura para expandir os pensamentos, provavelmente haverá mais possibilidades para embasar as escolhas. Nesse caminho, implica dar mais abertura para lidar com o diferente e repensar aspectos preconceituosos e discriminatórios. Portanto, é necessário criar esferas de diálogo compreensivo para as diversas demandas e construir um passo a passo de vivência intercultural e plural.

O cuidado de enfermagem em saúde da criança diante da referida temática necessita de ações que canalizem para a sabedoria prática, ou seja, que estejam atentas para entender atitudes e experiências, dar visibilidade às opiniões e dimensões ocultas, pormenorizar desejos, interesses, carências, esperanças e escolhas, bem como examinar as possibilidades de romper barreiras. Assim, volta-se para o desafio ético de enfermeiras/os para com o valor que a ação de saúde tem para os cuidadores parentais e suas crianças, construindo atitudes cuidadoras que incluam o sujeito como partícipe^(11)^.

O foco na diversidade, equidade e inclusão requer buscar transformações sociais e culturais para o cuidado, para reconhecer os marcadores identitários (gênero, orientação sexual, raça/cor de pele, etnia, religião, classe social, territorialidade, geração, deficiências, neurodiversidade, entre outros), respeitar as diferenças e trabalhar em prol das necessidades de saúde, da equidade e dos direitos humanos. Nos primeiros anos de vida, as interações das crianças com o ambiente físico e social, incluindo família, escola e comunidade, desempenham um papel determinante nas trajetórias de desenvolvimento, com implicações em longo prazo para a sua saúde, bem-estar e potencial futuro^(5)^.

Há implicações para o ensino, pesquisa e prática de enfermagem com a construção de competências de cunho interpessoal e intercultural, fundamentada em conhecimentos científicos, estratégias ativas e avanços permeados por novas situações e pelo encontro com o diferente, fundamentais na formação e educação permanente de enfermeiras/os, que necessitam de um arcabouço sólido para lidar com seres humanos, suas necessidades de saúde e características singulares, e para o trabalho em grupo e em equipes interprofissionais. Estudo destaca a necessidade de incorporar aos currículos de enfermagem os princípios de diversidade, equidade e inclusão, bem como a compreensão ampliada de como o preconceito interfere no cuidado e na promoção da saúde, buscando a desconstrução de preconceitos por meio da educação reflexiva^(25)^.

O cuidado de enfermagem com destaque ao seguimento pré-natal e de puericultura, em consulta, visita domiciliar e grupos educativos, comporta muitos elementos para a atenção integral à saúde da criança junto aos cuidadores parentais para a parentalidade positiva. Há também a relevância de enfatizar as ações intersetoriais entre saúde, educação e proteção social, que são setores de importantes interfaces para viabilizar a boa gestão do cuidado em contribuição ao desenvolvimento humano.

O cuidado profissional ofertado nesses encontros para a atenção à saúde infantil possibilita um conjunto de interações com as famílias. Para interações efetivas e responsivas às vivências das famílias, propõe-se que as boas práticas devem incluir a compreensão de que crenças e escolhas parentais são indispensáveis ao cuidado de enfermagem em saúde da criança, como parte de um repertório que busca obter o diferente e o diverso e dar vazão para emergir os saberes das experiências, interesses comuns ou divergentes, tensões, possibilidades e expectativas.

Assim, no âmbito da promoção do desenvolvimento infantil, esclarecer e fortalecer as escolhas parentais envolvendo as temáticas da diversidade, equidade e inclusão fazem parte do cuidado, para que sejam mais vinculadas à importância do desenvolvimento pleno das crianças com aprendizado e experiências diversas e multiculturais desde os primeiros anos de vida, e que as percepções parentais migrem de um rol de saberes de invisibilidade para expandir seus saberes práticos.

## CONSIDERAÇÕES FINAIS

O presente estudo destacou elementos exploratórios e reflexivos sobre escolhas parentais vinculadas ao cuidado da criança, apontando dilemas contemporâneos sobre a diversidade social em situações relativas ao cuidado exercido por alguém de outra cor de pele, outra orientação sexual, outra religião e figura masculina. As reflexões sobre saberes práticos foram enfatizadas para a promoção do desenvolvimento infantil, ressaltando a importância da enfermagem em lidar com experiências parentais, suas escolhas e supostas resistências às aptidões de outras pessoas para cuidar da criança.

A temática da diversidade social envolvendo as pessoas que exercem o cuidado da criança abrange desafios e oportunidades em busca do desenvolvimento pleno das crianças, que promovam a socialização, o desenvolvimento de conexões e o sentimento de pertencimento. Assim, é preciso colocar mais valor nas habilidades socioemocionais, cognitivas, de atenção, memória, capacidade de resolução de problemas e juízo crítico, necessárias ao longo da vida.

O cuidado de enfermagem em saúde da criança diante de aspectos referentes à diversidade social necessita expandir e buscar conhecer valores e caminhos para uma convivência humana satisfatória, evitando discriminação, preconceitos, hostilidade e conflitos, repensando e reconstruindo as relações, as ações e os compromissos. Portanto, no cuidado integral à saúde da criança, conhecer crenças e escolhas e incrementar a comunicação constituem um imperativo dos tempos contemporâneos. Pesquisas são necessárias para compreender as percepções dos cuidadores parentais e identificar o impacto de características sociais de cuidadores de crianças na promoção do desenvolvimento infantil.

## DISPONIBILIDADE DE DADOS

Todo o conjunto de dados que dá suporte aos resultados deste estudo foi publicado no próprio artigo.

# Translation: Diversidad social, parentalidad y promoción del desarrollo infantil: reflexiones sobre el cuidado de enfermería

**DOI:** 10.1590/1980-220X-REEUSP-2025-0208es

**Authors:** Carolina Beltreschi Bardivia, Maria de La Ó Ramallo Veríssimo, Elsa Maria de Oliveira Pinheiro de Melo, Débora Falleiros de Mello

**Affiliations:** 1 Universidade de São Paulo, Escola de Enfermagem de Ribeirão Preto, Programa de Pós-Graduação Enfermagem em Saúde Pública, Ribeirão Preto, SP, Brasil.; 2 Universidade de São Paulo, Escola de Enfermagem, São Paulo, SP, Brasil.; 3 Universidade de Aveiro, Escola Superior Saúde de Aveiro, Portugal.; 4 Universidade de São Paulo, Escola de Enfermagem de Ribeirão Preto, Ribeirão Preto, SP, Brasil.

**Keywords:** Cuidado del Niño, Desarrollo Infantil, Cuidadores, Responsabilidad Parental

## Abstract

**Objetivo:**

Enumerar elementos reflexivos sobre el cuidado infantil ante los dilemas contemporáneos que involucran la parentalidad y la diversidad social para promover el desarrollo infantil y sus implicaciones para la enfermería.

**Método:**

Estudio reflexivo desde la perspectiva teórica de la sabiduría práctica, apoyada en la comprensión del conocimiento práctico que implica elecciones frente a las contingencias cotidianas.

**Resultados:**

Se enumeraron elementos reflexivos sobre las opciones parentales y los posibles dilemas contemporáneos en torno a la diversidad social relacionada con el cuidado brindado por alguien de otro color de piel, otra orientación sexual, otra religión y figuras masculinas. El conocimiento práctico relacionado con las elecciones y la supuesta resistencia parentales a las capacidades de otras personas para cuidar a los niños sugiere ampliar las reflexiones sobre las implicaciones para la parentalidad y la promoción del desarrollo infantil.

**Conclusión:**

La sabiduría práctica contribuye a analizar y considerar aspectos del cuidado, aportando elementos importantes para que la enfermería pueda auxiliar en las decisiones de los padres que contemplen la diversidad social en la infancia, considerando evitar la discriminación, los prejuicios y la hostilidad, para valorar las oportunidades de promover el pleno desarrollo infantil.

## INTRODUCCIÓN

Los cuidados que los cuidadores primarios brindan a los niños son cruciales para promover el desarrollo infantil, con énfasis en la interacción receptiva que proporciona afecto y aprendizaje, especialmente durante los primeros años de vida^(1)^. En nuestra realidad, los cuidados primarios se brindan principalmente en el ámbito familiar, por parte de las madres, los padres o las personas que ejercen la función parental. La evaluación de la eficacia de las intervenciones parentales en el desarrollo infantil señala que las acciones de promoción son particularmente importantes en los primeros tres años de vida, cuando el cerebro en desarrollo es más sensible a las experiencias y al entorno^(1)^.

La parentalidad se entiende como el conjunto de estrategias llevadas a cabo por los cuidadores con función parental, con el fin de promover el desarrollo del niño, garantizar su seguridad y proporcionar los estímulos adecuados^(2)^.

Los cuidadores parentales tienen muchas tareas que realizar para cuidar y comprender información que puede ser compleja, nueva, específica o incierta, por lo que es esencial contar con personas de referencia que puedan apoyar los conocimientos y las prácticas parentales sobre el desarrollo infantil y sus beneficios para el cuidado integral del niño^(3)^.

En el cuidado de los niños, también son de suma importancia los cuidadores no parentales, incluidos los familiares, las niñeras y los educadores de guarderías, que pueden ser cuidadores primarios o secundarios^(4)^. La promoción del desarrollo integral del niño incluye aspectos de socialización, que implican el aprendizaje de la convivencia entre pares y en la comunidad, formas de interacción, empatía, respeto y alteridad, optimizando las trayectorias de desarrollo de los niños^(5)^.

En este proceso, es necesario prestar atención a la pertenencia al grupo, la exposición a la diversidad y las interacciones sociales plurales. El cuidado parental y no parental puede tener implicaciones en cuestiones de diversidad social, incluyendo la discriminación por motivos de racismo^(6)^, la hostilidad relacionada con la diversidad por orientación sexual^(7)^, obstáculos para la aceptación de diferentes religiones^(8)^ y dificultades para una mayor participación de la figura masculina en el cuidado infantil^(9)^, que han sido señaladas en diferentes contextos sociales por sus repercusiones en el desarrollo de los niños. En particular, las instituciones de educación infantil deben garantizar la estimulación social y cognitiva en un entorno seguro y con calidad en las acciones ofrecidas^(10)^.

Ante las evidencias científicas que destacan la importancia de los cuidados que se ofrecen a los niños, con énfasis en la interacción receptiva y el papel de la parentalidad positiva, y también ante los dilemas contemporáneos a la hora de lidiar con las diferencias, la discriminación y la intolerancia, así como la necesidad de incrementar las acciones inclusivas, este estudio busca discutir aspectos relacionados con la parentalidad y la diversidad social y sus posibles repercusiones en la promoción del desarrollo infantil. Así, su objetivo es enumerar elementos reflexivos sobre el cuidado del niño frente a los dilemas contemporáneos que involucran la parentalidad y la diversidad social para la promoción del desarrollo infantil y sus implicaciones para la enfermería.

## REFERENCIAL TEÓRICO-METODOLÓGICO

Estudio reflexivo desde la perspectiva de la sabiduría práctica^(10)^, en busca de comprender los conocimientos prácticos relacionados con la parentalidad y la diversidad social en pro de la promoción del desarrollo infantil. En este sentido, se plantea para la reflexión el cuidado centrado en la diversidad social en relación con el cuidado infantil ejercido por alguien de otro color de piel, otra orientación sexual, otra religión y/o por figuras masculinas.

En el presente estudio, se aborda la perspectiva de la sabiduría práctica para el cuidado de enfermería en la salud infantil, destacando la importancia de estar en consonancia con las realidades contextualizadas y compartidas entre los cuidadores parentales y profesionales, y en busca de valorar las experiencias, las elecciones y la toma de decisiones para comprender y ampliar el cuidado en salud^(11)^.

La sabiduría práctica es un concepto originario de la filosofía práctica, en el ámbito de los conocimientos aristotélicos sobre las racionalidades del saber^(10)^. El plano de la sabiduría práctica implica una especificidad contingente, es decir, se ocupa de la eventualidad, las incertidumbres, los acontecimientos y las experiencias humanas, reconociendo que los aspectos interrelacionados no son perennes, causales y universales^(10)^. En este sentido, constituye un espacio de conocimiento con capacidad para que surjan intereses comunes o divergentes, valores y posibilidades de interacción y diálogo.

La perspectiva teórica de la sabiduría práctica implica los conocimientos prácticos relacionados con los procesos de elección (lograr elegir, precisar elegir, necesitar saber y encontrar lo bueno en cada situación vivida) y el concepto de contingente como su esencia^(10)^. Por lo tanto, se trata también de un conjunto de conocimientos que tratan con menos certeza y determinación, y buscan construir la comprensión de aspectos de la vida, las experiencias y las elecciones ante las diversas contingencias de la vida cotidiana.

Los conocimientos prácticos y sus interfaces con los conocimientos técnico-científicos se refieren a un movimiento de reconstrucción y articulación de diferentes aspectos que componen un tema o una situación, y que pueden ayudar al proceso de las elecciones humanas^(10)^. En el caso de la atención a la salud y el desarrollo infantil y su relación con la parentalidad, las decisiones de los padres son muy importantes. Para ello, son vitales la comunicación, el lenguaje, la escucha y el diálogo entre los sujetos, a fin de fomentar buenas decisiones de cuidado y reflexionar críticamente sobre las preocupaciones y dilemas de percepciones y perspectivas, y que contribuyan a tener más claridad hacia las buenas prácticas.

En el campo de la promoción del desarrollo infantil, los profesionales pueden basarse en la sabiduría práctica, es decir, prestar atención a las experiencias de los cuidadores parentales y sus elecciones frente a las necesidades de cuidado de sus hijos, lo que implica la responsabilidad de adoptar actitudes de cuidado pertinentes.

## PARENTALIDAD Y PROMOCIÓN DEL DESARROLLO INFANTIL: UNA MIRADA A LA DIVERSIDAD SOCIAL

El conjunto de experiencias que el niño vive en los primeros años de vida constituye un repertorio de gran relevancia en la formación del cerebro y en la construcción de aprendizajes y habilidades motoras, cognitivas, emocionales y sociales. Por lo tanto, todas las interacciones y entornos deben actuar sinérgicamente con cuidados y estímulos adecuados para proporcionar un buen desarrollo^(1)^.

Los horizontes del cuidado parental y no parental desempeñan un papel significativo en la vida de los niños y, en particular, las situaciones de quienes cuidan al niño y el entorno en el que se inserta en los primeros años de vida deben componer el diálogo con las familias y favorecer las buenas decisiones parentales. Cuando el cuidado del niño se considera desde la perspectiva de proporcionar su pleno desarrollo, también cobran relevancia los cuidados no parentales prestados por otras personas. En este sentido, son importantes las oportunidades de convivencia con alguien de otro color de piel, otra orientación sexual, otra religión o figuras masculinas.

En lo que respecta a los aspectos relacionados con el color de la piel y el desarrollo infantil, un estudio analiza el papel de los padres en el abordaje de los prejuicios^(6)^. Señala que los niños pueden comenzar a mostrar prejuicios raciales en las primeras etapas del desarrollo infantil, por lo que es necesario prevenir y reducir los aspectos que puedan implicar prejuicios. Las cuestiones de discriminación relacionadas con el racismo pueden ser una barrera para la atención médica. El estudio encontró que las actitudes raciales de los padres que niegan los privilegios de los blancos se relacionaban negativamente con la simpatía de los niños hacia las víctimas negras, abordando las formas potenciales de moldear la simpatía y la compasión de los niños en función de la raza^(12)^. Los bebés que crecieron en comunidades más diversas mostraron una mayor capacidad para reconocer rostros de otras etnias, en comparación con los que vivieron en comunidades menos diversas^(13)^. Por lo tanto, la exposición a una variedad de rostros desde la primera infancia parece desempeñar un papel crucial en la aceptación de la diversidad racial a lo largo de la vida.

En relación con los aspectos de la orientación sexual, las cuestiones relacionadas con la diversidad sexual pueden desencadenar una serie de actitudes de hostilidad, marginación y exclusión. En el cuidado de los niños, es importante el debate sobre la orientación sexual, la parentalidad y el bienestar de los niños^(7)^. Este estudio hace hincapié en que no se deben hacer suposiciones basadas en la orientación sexual de las personas, si son más adecuadas que otras para cuidar a los niños^(7)^.

Otro aspecto a destacar se refiere a los obstáculos para la aceptación de diferentes religiones, ya que la intolerancia religiosa puede afectar negativamente al desarrollo y la autoestima humana^(8)^. Prestar atención a las conexiones entre la religión y las actitudes culturales específicas, por ejemplo, en relación con los inmigrantes, los ateos y los grupos religiosos minoritarios, cobra importancia para la inclusión de enfoques interdisciplinarios y centrados en la persona^(8)^. La forma en que las familias y las escuelas abordan la convivencia con diferentes religiones puede influir en la aceptación o el rechazo de creencias distintas, y la manera en que los educadores abordan la diversidad religiosa contribuye a promover una convivencia respetuosa^(14,15)^.

En cuanto a la presencia activa de la figura masculina en el cuidado infantil, las implicaciones en lo que respecta a ayudar a los niños a comprender que el cuidado no es una responsabilidad exclusiva de las mujeres y las niñas son muy importantes, además de permitir comprender que los hombres pueden ser cuidadores, lo que contribuye a la construcción de relaciones afectivas más equilibradas^(16)^. De este modo, la deconstrucción de los estereotipos promueve una visión más igualitaria y colaborativa entre hombres y mujeres^(16)^.

La figura paterna que desea involucrarse más en el cuidado de los niños puede enfrentarse a barreras culturales, falta de recursos y un apoyo inadecuado, lo que pone de relieve la necesidad de desmitificar la imagen de la paternidad tradicional. Así, muchos hombres siguen siendo socializados con la expectativa de convertirse en el proveedor financiero, y la madre es vista como la principal responsable del niño^(17)^. El cuidado de los niños, visto como una función secundaria, puede perpetuar los roles tradicionales de fuerza y autoridad, y este estigma puede generar inseguridad y dificultar la participación paterna en el cuidado pleno de los niños^(18)^.

En la educación infantil, la figura masculina se considera beneficiosa, ya que promueve una formación plural, además de desempeñar un papel crucial en el desarrollo integral del niño, especialmente en lo que se refiere a la construcción de la identidad y el desarrollo de habilidades cognitivas y socioemocionales^(19)^. En este sentido, ampliar el potencial de participación de las figuras masculinas en el cuidado diario del niño contribuye a estimular un enfoque transformador del género^(9)^.

Las oportunidades que proporcionan las actitudes intergrupales de género de los niños tienen implicaciones para el comportamiento entre pares, como lo demuestra el estudio con niños de entre 4 y 5 años de edad de diversos orígenes (mexicanos, chinos, dominicanos y afroamericanos), en el que las actitudes positivas hacia el mismo sexo se asociaron con el conocimiento de los estereotipos de género y, por el contrario, las actitudes hacia otros géneros se asociaron con la versatilidad cognitiva (flexibilidad de estereotipos, consistencia de género), y las actitudes de otro género predijeron un comportamiento prejuicioso^(20)^. El aprendizaje temprano sobre las categorías de género sugiere moldear las actitudes de los niños pequeños, con consecuencias para el comportamiento intergrupal en la infancia^(20)^.

En situaciones de cuidados no parentales en la infancia, cuando el cuidado lo realiza alguien de otro color de piel, otra orientación sexual, otra religión y/o figura masculina, hay que prestar atención a la relación entre los cuidadores parentales y no parentales. En este sentido, es fundamental dar prioridad a las interacciones y al entorno de cuidado, para que contribuyan al desarrollo infantil, independientemente de las características personales y sociales. Por lo tanto, es necesario prestar atención a la implicación con el niño, al potencial de contribuir a su desarrollo y a las percepciones y hechos sobre los impactos futuros. También es importante prestar atención a los ciclos globales de pobreza, desigualdad y exclusión social, que generan dificultades para alcanzar el potencial de desarrollo humano^(21)^.

Estos aspectos cobran importancia en la comprensión de la sabiduría práctica, que implica las percepciones y elecciones de los padres y el cuidado integral de los niños, con el fortalecimiento de las iniciativas de interacciones positivas entre el niño y el entorno y la promoción del desarrollo en la primera infancia.

Es esencial promover entornos equitativos e inclusivos para los niños, así como enfoques que fomenten actitudes positivas para abordar la diversidad, ya que configuran oportunidades para facilitar la socialización, el desarrollo de conexiones y un sentimiento de pertenencia^(22)^. Cabe destacar la necesidad de una buena composición de los primeros entornos de cuidado infantil, con relaciones sociales oportunas, herramientas pertinentes para analizar la profundidad y amplitud de las relaciones, y redes sociales a lo largo del desarrollo^(23)^. Así, la categorización social se va construyendo y tiene sus raíces en sistemas perceptivos, conceptuales y sociales que surgen desde una edad temprana y experimentan importantes cambios de desarrollo durante la infancia, con posibles repercusiones a lo largo de la vida^(24)^.

La base de la capacidad humana para formar categorías sociales útiles se produce en la infancia, lo que permite orientar las inferencias que los niños hacen a partir de las características y relaciones sociales compartidas^(21)^. Para los niños, la categorización social puede ayudar a simplificar y comprender su entorno social, pero tiene consecuencias perjudiciales en forma de estereotipos, prejuicios y discriminación^(24)^. En este sentido, la educación infantil se considera una de las formas de combatir los prejuicios relacionados con la diversidad y promover una mejor inclusión, siempre que los entornos proporcionen una estimulación social y cognitiva cualificada^(10)^. A nivel mundial, se han recomendado estrategias cualitativas e inclusivas en la educación infantil para alcanzar las metas de los Objetivos de Desarrollo Sostenible, incluidos los dirigidos a promover el desarrollo infantil^(22)^.

En el presente estudio, los elementos reflexivos más destacados se refieren a las perspectivas que el cuidado parental puede tener en la promoción del desarrollo infantil, en el que una gama de experiencias parentales y no parentales es muy relevante, siendo vitales los aspectos que atraviesan las elecciones, consideraciones, argumentos y conocimientos reflexivos. Las dimensiones de las elecciones relacionadas con la diversidad social pueden contener obstáculos y desafíos o estar abiertas a romper con la intolerancia, la discriminación y los prejuicios. En este sentido, conviene privilegiar el diálogo entre los sujetos involucrados para ampliar los entendimientos y redefinir el cuidado del niño y su desarrollo, que son relevantes para la atención sanitaria y de enfermería en este campo.

Por lo tanto, esta reflexión no pretende abordar ni debatir definiciones específicas de diversidad étnica, racial, cultural, religiosa, de género y de orientación sexual. La perspectiva es reflexionar sobre el cuidado de enfermería frente a los retos que implican estos aspectos para la promoción del desarrollo humano en la infancia.

### Contribuciones al Campo de la Enfermería

Las observaciones relativas al cuidado de los niños por parte de otras personas aportan aspectos contemporáneos importantes para repensar la atención de enfermería que implica la atención sanitaria en la primera infancia. El proceso de cuidado de los niños, impregnado de las decisiones de los padres, abarca el tema de la diversidad social, por lo que es importante dar espacio a las reflexiones sobre las interfaces con las oportunidades de desarrollo integral del niño y la expansión de la sabiduría práctica.

Cuidar de la salud de los niños junto con los cuidadores parentales requiere competencias para ampliar las articulaciones con los conocimientos prácticos^(11)^. El tema de la diversidad social en el cuidado infantil implica abarcar y superar el éxito técnico, alcanzando actitudes comprensivas en el proceso de cuidado y reconstruyendo los entendimientos sobre las situaciones que viven las familias y cómo manejan los recursos disponibles en la comunidad.

La elección de los padres sobre quién cuidará al niño es, en cierto modo, voluntaria, pero si hay más información, diálogo conjunto y apertura para ampliar las ideas, probablemente habrá más posibilidades para fundamentar las elecciones. En este sentido, implica dar más apertura para lidiar con lo diferente y replantearse aspectos prejuiciosos y discriminatorios. Por lo tanto, es necesario crear esferas de diálogo comprensivo para las diversas demandas y construir paso a paso una experiencia intercultural y plural.

La atención de enfermería en salud infantil ante esta temática requiere acciones que canalicen hacia la sabiduría práctica, es decir, que estén atentas a comprender actitudes y experiencias, dar visibilidad a opiniones y dimensiones ocultas, detallar deseos, intereses, carencias, esperanzas y elecciones, así como examinar las posibilidades de romper barreras. Así, se vuelve al desafío ético de las enfermeras/os con respecto al valor que la acción de salud tiene para los cuidadores parentales y sus niños, construyendo actitudes de cuidado que incluyan al sujeto como participante^(11)^.

El enfoque en la diversidad, la equidad y la inclusión requiere buscar transformaciones sociales y culturales para el cuidado, reconocer los marcadores de identidad (género, orientación sexual, raza/color de piel, etnia, religión, clase social, territorialidad, generación, discapacidades, neurodiversidad, entre otros), respetar las diferencias y trabajar en pro de las necesidades de salud, la equidad y los derechos humanos. En los primeros años de vida, las interacciones de los niños con el entorno físico y social, incluyendo la familia, la escuela y la comunidad, desempeñan un papel determinante en las trayectorias de desarrollo, con implicaciones a largo plazo para su salud, bienestar y potencial futuro^(5)^.

La construcción de competencias interpersonales e interculturales, basadas en conocimientos científicos, estrategias activas y avances impregnados de nuevas situaciones y del encuentro con lo diferente, tiene implicaciones para la enseñanza, la investigación y la práctica de la enfermería, fundamentales en la formación y la educación permanente de las enfermeras, que necesitan un marco sólido para tratar con los seres humanos, sus necesidades de salud y sus características singulares, y para el trabajo en grupo y en equipos interprofesionales. El estudio destaca la necesidad de incorporar en los planes de estudio de enfermería los principios de diversidad, equidad e inclusión, así como una comprensión más amplia de cómo los prejuicios interfieren en la atención y la promoción de la salud, buscando la deconstrucción de los prejuicios a través de la educación reflexiva^(25)^.

Los cuidados de enfermería, con especial énfasis en el seguimiento prenatal y la puericultura, en consultas, visitas domiciliarias y grupos educativos, comprende muchos elementos para la atención integral de la salud del niño junto con los cuidadores parentales para una parentalidad positiva. También es importante destacar las acciones intersectoriales entre salud, educación y protección social, que son sectores de importantes interfaces para viabilizar la buena gestión de la atención en contribución al desarrollo humano.

Los cuidados profesionales ofrecidos en estos encuentros para la atención de la salud infantil permiten un conjunto de interacciones con las familias. Para que las interacciones sean efectivas y respondan a las experiencias de las familias, se propone que las buenas prácticas incluyan la comprensión de que las creencias y las decisiones de los padres son indispensables para la atención de enfermería en salud infantil, como parte de un repertorio que busca obtener lo diferente y lo diverso y dar rienda suelta a los conocimientos de las experiencias, los intereses comunes o divergentes, las tensiones, las posibilidades y las expectativas.

Así, en el ámbito de la promoción del desarrollo infantil, aclarar y fortalecer las decisiones de los padres en torno a los temas de la diversidad, la equidad y la inclusión forman parte de la atención, para que estén más vinculadas a la importancia del pleno desarrollo de los niños con aprendizajes y experiencias diversas y multiculturales desde los primeros años de vida, y que las percepciones de los padres pasen de un rol de conocimientos invisibles a ampliar sus conocimientos prácticos.

## CONSIDERACIONES FINALES

El presente estudio destacó elementos exploratorios y reflexivos sobre las decisiones de los padres relacionadas con el cuidado de los niños, señalando dilemas contemporáneos sobre la diversidad social en situaciones relacionadas con el cuidado ejercido por alguien de otro color de piel, otra orientación sexual, otra religión y/o figura masculina. Se enfatizaron las reflexiones sobre los conocimientos prácticos para promover el desarrollo infantil, destacando la importancia de la enfermería en el manejo de las experiencias parentales, sus elecciones y supuestas resistencias a las aptitudes de otras personas para cuidar al niño.

El tema de la diversidad social que involucra a las personas que cuidan a los niños abarca desafíos y oportunidades en la búsqueda del pleno desarrollo de los niños, que promuevan la socialización, el desarrollo de conexiones y el sentimiento de pertenencia. Por lo tanto, es necesario valorar más las habilidades socioemocionales, cognitivas, de atención, memoria, capacidad de resolución de problemas y juicio crítico, necesarias a lo largo de la vida.

El cuidado de enfermería en la salud infantil ante aspectos relacionados con la diversidad social necesita expandirse y buscar conocer valores y caminos para una convivencia humana satisfactoria, evitando la discriminación, los prejuicios, la hostilidad y los conflictos, replanteando y reconstruyendo las relaciones, las acciones y los compromisos. Por lo tanto, en la atención integral a la salud infantil, conocer las creencias y elecciones e incrementar la comunicación constituyen un imperativo de los tiempos contemporáneos. Se necesitan investigaciones para comprender las percepciones de los cuidadores parentales e identificar el impacto de las características sociales de los cuidadores de niños en la promoción del desarrollo infantil.

## DISPONIBILIDAD DE DATOS

Todo el conjunto de datos que apoya los resultados de este estudio fue publicado en el propio artículo.
